# Responses to criticisms

**DOI:** 10.1007/s44204-025-00302-x

**Published:** 2025-07-21

**Authors:** Yafeng Shan, Jon Williamson

**Affiliations:** 1 Division of Humanities and Center for Philosophy of Science, The Hong Kong University of Science and Technology, Clear Water Bay, Hong Kong, China; 2 Department of Philosophy, University of Manchester, Manchester, UK

**Keywords:** Evidential pluralism, Social science, Transitivity, Mixed methods research, Knowledge-first, Establish

## Abstract

Responding to the critical commentaries by Rosa Runhardt, Erik Weber, and Michael Wilde, we defend the application of Evidential Pluralism to the social sciences.

We are very grateful to Rosa Runhardt, Erik Weber and Michael Wilde for their detailed comments and criticisms of our book, *Evidential Pluralism in the Social Sciences. *(Shan and Williamson 2023) We focus here on responding to what we take to be their main criticisms.

## Response to Rosa W. Runhardt

In her paper ‘Evidential pluralism in the social sciences: What can be established in case study research?’ Runhardt (2024) challenges Evidential Pluralism by putting forward a putative counterexample in which a causal claim can be established on the basis of evidence of mechanisms only—no correlation is required.

Runhardt’s example proceeds as follows. The causal claim is that ‘the election of President Joe Biden in 2020 caused the continuation of Title 42 immigration rule in 2021 (i.e., the continuation of the controversial pandemic-era rule allowing the U.S. to rapidly expel asylum seekers and migrants, especially at the U.S.-Mexico border, citing a public health emergency)’. Runhardt suggests that a mechanism can be found that links:

(B) The election of President Joe Biden in 2020.

to

(C) The continuation of the Title 42 immigration rule in 2021.

Runhardt maintains, however, that there is no positive correlation between B and C: in fact, B lowered the chance of C. If this is the case, and if it is indeed established that B caused C, then Evidential Pluralism is undermined, because Evidential Pluralism requires both correlation and mechanism to be established when establishing a causal claim.

We would deny that this provides a genuine counterexample to Evidential Pluralism, however, because we would deny that B caused C.

For the sake of argument, let us grant the premisses of the counterexample:

1. There is a mechanism from B to C.
2. B in fact lowered the chance of C.

Runhardt wants to appeal to (1) to say that B caused C. But given (2), it seems awkward to say that B caused C: one would normally say that C happened *despite* B, not that B caused C.

This is essentially a debate about the transitivity of causation (see, e.g. Menzies & Beebee, 2024, Section 2.2). Note that transitivity is a universal claim: proponents of transitivity maintain that in every single case in which there is a chain of causal relationships linking a sequence of events, the first event in the chain must be classified as a cause of the last. Those who deny transitivity maintain that while in some such cases it is appropriate to deem the first event to be a cause of the last, in other cases, it is not.

We deny the transitivity of causation. Indeed, this example illustrates the failure of transitivity. Plausibly, some decision D to continue the Title 42 immigration rule caused C, and plausibly, there is also a chain of events from B to D, resulting in a chain of events from B to C, in line with premiss (1). This is not enough on its own to warrant the claim that C caused D, however. Indeed, B should not be construed as having caused C, because B lowered the chance of C (premiss 2). We would say that in this example, the chain of events from B to C underpins a *narrative explanation* of C, but not a *causal explanation* (see p. 126 of our book).

So, we maintain that B is not a cause of C while Runhardt maintains that it is. Are there any further resources to which we can appeal in order to adjudicate this disagreement? We introduce the epistemic theory of causality in Section 5 of our book, and the epistemic theory maintains that the characteristic feature of causal relationships is that they support prediction, explanation and control (‘PEC’) inferences. At the end of Section 3 of her response, Runhardt suggests that PECs would support her analysis of the example: i.e. Runhardt suggests that one can draw the appropriate prediction, explanation and control inferences, so the relationship between B and C must be causal.

But this seems straightforwardly false. Given (2), it would not have been reasonable to predict that the immigration rule would continue with Biden elected. Indeed, it was a surprise that the rule continued. Consequently, B is not predictive of C. Moreover, the relationship from B to C does not support a control inference: it would not have been reasonable to vote for Biden in order to ensure the continuation of the rule. Given that B lowered the chance of C, attempting to bring about B would at the time have been a very bad strategy for ensuring C.

So, although there is some sort of explanation of C that involves B, the relationship between B and C does not support the prediction and control inferences that one would expect if B were a cause of C. Hence B should not be classified as a cause of C, and Runhardt’s example is not a genuine counterexample to Evidential Pluralism.

## Response to Erik Weber

Weber (2025), in his paper ‘Evidential pluralism, epistemic causality and mixed methods research’, compares the epistemic theory of causality to Giere’s difference-making analysis of causation. Weber suggests that Giere’s analysis is not susceptible to our objections to difference-making theories of causation because it does not analyse causality in terms of correlation. Furthermore, Weber claims that Giere’s account is not incompatible with Evidential Pluralism. We shall challenge this claim.

Giere’s theory holds that:

C is a cause of E iff the probability of E were all individuals to exhibit C would differ from the probability of E were no individuals to exhibit C.

This kind of probabilistic dependence is not usually thought of as a correlation, because of its counterfactual nature. We shall see, however, that this account suffers from the same problem that we argue faces other difference-making accounts of causality: it fails to validate the rationality of seeking evidence of mechanisms when a correlation has been established (see p. 25 of our book).

According to Giere’s analysis, in order to establish that smoking is a cause of cancer, we would need to establish what would happen if all individuals smoked and what would happen if no individuals smoked. What do we need to do to establish these counterfactual claims? Giere (1979, Chapters 7–8) argues that we need to carry out association studies; he offers no discussion of mechanistic evidence.

Weber maintains that we can extend Giere’s account by using Evidential Pluralism as a more comprehensive account of the epistemology of causality (Weber, 2009). But Evidential Pluralism is hard to square with Giere’s analysis. Giere’s analysis suggests that to judge whether C is a cause of E, we need to calculate two (counterfactual) probabilities and compare them. These need to be accurate calculations, not rough estimates, because we need to decide whether or not the two probabilities are equal. Evidential Pluralism does not tell us how to calculate these probabilities—it tells us how to evaluate causality without calculating these probabilities. Thus, those who argue that Giere’s analysis can accommodate Evidential Pluralism owe us an account of how to calculate these probabilities without relying solely on estimates from association studies. Without such an account, it seems that Giere’s analysis would fail to validate the rationality of seeking evidence of mechanisms when the existence of a correlation has been established. Thus, we see that Giere’s theory of causality is not immune to our objections to difference-making theories of causality.

This concern is not merely theoretical—it also casts doubt on the claim that Giere’s analysis of causation can accommodate real examples of causal enquiry in the social sciences. Consider Donohue and Levitt’s famous study of crime rates in the USA (2001), discussed in Section 24 of our book. Donohue and Levitt argue that the legalisation of abortion in the 1970s was a cause of the decline in the crime rates in the 1990s in the USA. On the one hand, Donohue and Levitt show that the legalisation of abortion was strongly associated with a subsequent drop in crime by conducting regression analyses of: crime and abortion rates in national time series; differential crime patterns between states which legalised abortion early on and those that legalised abortion later on; and state abortion and crime rates. On the other hand, Donohue and Levitt also provide evidence for two mechanisms linking the legalisation of abortion in the early 1970s and the decline in the crime rates in the 1990s. Giere’s analysis seems to validate Donohue and Levitt’s use of association studies, since these studies provide some support for the claim that the probability of a reduction in crime rates were all states to legalise abortion differs from the probability of a reduction in crime rates were no states to legalise abortion. But if Giere’s theory were correct, there would seem to be no further need to conduct mechanistic studies to unpack the ‘theoretical link between legalization of abortion in the early 1970s and subsequent drops in crime fifteen to twenty years later’ (Donohue & Levitt, 2001, p. 386). The concern is that Giere’s analysis fails to account for the use of mechanistic studies where there is already good evidence of correlation.

Weber goes on to discuss the foundations of mixed methods research. Evidential Pluralism offers strong foundations for mixed methods research: while association studies normally employ quantitative methods, mechanistic studies can employ quantitative or qualitative methods; so, since Evidential Pluralism says that we need to scrutinise both association studies and mechanistic studies when evaluating causation, we had better scrutinise both quantitative and qualitative studies (see chapter 4 of our book). Weber argues, however, that alternative foundations for mixed methods research can be provided by separating the task of *establishing* a causal relationship from that of *understanding*/*explaining* the relationship. Weber’s idea is very roughly that quantitative methods are needed to establish the relationship and that qualitative methods for exploring the underlying mechanisms are needed to help us understand/explain the relationship.

We would respond to this point in two ways.

First, we should clarify that our argument is that Evidential Pluralism provides strong foundations for mixed methods research—not that *only* Evidential Pluralism can provide strong foundations for mixed methods research. We do not deny that there may be other philosophical theories which provide strong foundations for mixed methods research. Thus, even if it were successfully shown that an alternative philosophical theory provides strong foundations for mixed methods research, that would not undermine our argument about Evidential Pluralism and mixed methods research.

Second, we would argue that Weber’s proposed alternative in fact fails to motivate mixed methods research. Weber conceives of a division of labour which, we would argue, motivates two kinds of single-method research rather than mixed methods research. According to Weber, one kind of research (the use of quantitative methods) applies to the task of establishing and the other kind (qualitative methods) to the task of understanding/explaining. This does not motivate the use of mixed methods research for either task. That you need a hammer to fix a nail and a brush to paint a picture does not imply that you need a hammer-brush mixture for any specific task.

Evidential Pluralism, in contrast, does motivate the need for a mixture of methods for some specific task: if Evidential Pluralism is correct, we need to scrutinise both quantitative and qualitative studies for the task of evaluating a causal claim. This is because we need to scrutinise both association studies and mechanistic studies, and while quantitative methods are crucial to the former, qualitative methods are often essential to the latter. As Weber himself acknowledges,

the traditional story of adherents of mixed methods research has a blind spot that is revealed and remediated by evidential pluralism: they emphasise the role of mechanisms in explanation at the expense of the evidential role of knowledge of mechanisms. (Weber, 2025, pp. 8–9).

## Response to Michael Wilde

Evidential Pluralism is a theory of how to establish and evaluate causal claims. When introducing Evidential Pluralism, we set out what we mean by ‘establish’ (pp. 4–6 of our book). In ‘Evidential Pluralism and accounts of establishing’, Wilde (2025) argues that an alternative, non-evidentialist account of establishing is also compatible with the main claims of Evidential Pluralism. This is an interesting point, and we hope that it will help to make Evidential Pluralism attractive to those who hold different intuitions about what it is to establish a proposition.

Our approach was to try to achieve a balance between generality and concreteness when outlining Evidential Pluralism in chapter 1 of our book. On the one hand, presenting the view in very general terms allows a wide range of philosophers to embrace Evidential Pluralism, as Wilde suggests. On the other, we felt that it was incumbent upon us to show that there is at least one viable way to make the approach more concrete, in order to meet the concerns of potential sceptics and to make the subject more approachable to non-philosophers. Thus, we sought to show that Evidential Pluralism is compatible with at least one view of the metaphysics of causality—the epistemic theory of causality. Similarly, we sketched a view of establishing that we feel fits well with the way in which causal claims are established in the sciences.

Why did we not pick an account of establishing the kind that Wilde discusses? Because we felt that such an account has certain limitations (see, e.g. Williamson, 2015, §3) that would need addressing, and we did not want to get distracted from the main job at hand, namely introducing Evidential Pluralism. We are grateful to Wilde for addressing some of these limitations.

An important concern remains, however, for an account of establishing of the sort that Wilde discusses, which is based on the supposition that evidence is knowledge (E = K). The worry here is that evidence and knowledge seem to behave somewhat differently. On the one hand,

- (i) If you take *E* to be your evidence and you are rational to do so, then *E is* your evidence.

But on the other,

- (ii) You may take proposition *p* to be knowledge, and rationally so, yet be wrong about this.

This may be because you have extensive evidence, all of which is strongly in favour of *p*, but which unfortunately misleads you into inferring that *p* is true when it is not. Your strong evidence for *p* makes it rational to take *p* as knowledge, even though, *p* being false, it is not. Alternatively, (ii) could be down to sheer chance. If, as Wilde maintains, you can know *p* when there is a chance that *not-p*, then you can rationally take *p* to be knowledge when there is a chance that *not-p*, and indeed—that chance materialising—when *p* turns out to be false. *p*, being false, is not knowledge, so (ii) holds. (i) and (ii) lead to the conclusion that evidence and knowledge have different features, so E ≠ K.

Proponents of E = K will be most inclined to deny (ii). They may well say that you are only rational to take *p* as knowledge if *p* is in fact knowledge. This sort of response flies in the face of how we usually think of rationality, however. Plausibly, what is rational for you at any point in time depends only on information you possess at that time (Wedgwood, 2017, Section 2.4), and,

Whenever we assess any process of reasoning or mental state or event as rational or irrational, we are assessing it on the basis of its relation to the mental events and states that are present in the thinker’s mind—not on the basis of its relation to facts about the external world that could vary while those mental events and states remained unchanged. (Wedgwood, 2017, p. 163.)

Even advocates of E = K themselves tend to recognise these internalist features of our ordinary concept of rationality—see, e.g. Bird (2007), Section 6, and Williamson (2017, [Bibr CR14][Bibr CR13]). In particular, Williamson (2025b) argues that there are two concepts of rationality, one which corresponds to the above internalist notion and another that is externalist. (ii) would arguably fail under an externalist reading but not under an internalist reading. This appeal to two concepts of rationality does not undermine the objection to E = K, though. That evidence and knowledge behave differently with respect to one of these concepts (our ordinary internalist notion of rationality) is enough to imply that E ≠ K. Proponents of E = K will need to go further here—they will need to deny that evidence and knowledge behave differently with respect to our ordinary internalist concept of rationality. Most likely, they will deny that (i) holds under an internalist view of rationality. To deny (i), however, seems to us to be very counterintuitive.

The concern is thus that E = K conflicts with rationality, as ordinarily conceived. Perhaps, this concern can be alleviated. If so, so much the better that Evidential Pluralism is compatible with an account of establishing based on E = K. But if not, we can at least fall back on the account of establishing that we sketch in our book.

## Data Availability

No datasets were generated or analysed, due to the theoretical nature of this research.
